# CYLD Negatively Regulates Nontypeable *Haemophilus influenzae*-Induced IL-8 Expression via Phosphatase MKP-1-Dependent Inhibition of ERK

**DOI:** 10.1371/journal.pone.0112516

**Published:** 2014-11-12

**Authors:** Wenzhuo Y. Wang, Kensei Komatsu, Yuxian Huang, Jing Wu, Wenhong Zhang, Ji-Yun Lee, Masanori Miyata, Haidong Xu, Jian-Dong Li

**Affiliations:** 1 Center for Inflammation, Immunity & Infection, Institute for Biomedical Sciences, Georgia State University, Atlanta, Georgia, United States of America; 2 Department of Microbiology and Immunology, University of Rochester Medical Center, Rochester, New York, United States of America; 3 Department of Infectious Disease, Huashan Hospital, Fudan University, Shanghai, China; 4 Department of Viral Hepatitis, Shanghai Public Health Clinical Center, Fudan University, Shanghai, China; Rush University Medical Center, United States of America

## Abstract

Nontypeable *Haemophilus influenzae* (NTHi), a Gram-negative bacterium, is the primary cause of otitis media in children and the exacerbation of chronic obstructive pulmonary disease in adults. A hallmark of both diseases is an overactive inflammatory response, including the upregulation of chemokines, such as interleukin-8 (IL-8). An appropriate inflammatory response is essential for eradicating pathogens. However, excessive inflammation can cause host tissue damage. Therefore, expression of IL-8 must be tightly regulated. We previously reported that NTHi induces IL-8 expression in an ERK-dependent manner. We also have shown that the deubiquitinase cylindromatosis (CYLD) suppresses NTHi-induced inflammation. However, the underlying molecular mechanism of how CYLD negatively regulates ERK-mediated IL-8 production is largely unknown. Here, we examine both human lung epithelial A549 cells and lung of *Cyld* ^−/−^ mice to show that CYLD specifically targets the activation of ERK. Interestingly, CYLD enhances NTHi-induced upregulation of another negative regulator, MAP Kinase Phosphatase-1 (MKP-1), which, in turn, leads to reduced ERK activation and subsequent suppression of IL-8. Taken together, the CYLD suppression of ERK-dependent IL-8 via MKP-1 may bring novel insights into the tight regulation of inflammatory responses and also lead to innovative therapeutic strategies for controlling these responses by targeting key negative regulators of inflammation.

## Introduction

Nontypeable *Haemophilus influenzae* (NTHi) is an aerobic Gram-negative bacterium that is frequently found in the nasopharynx microbiome of the general population [1]. However, it is also an opportunistic pathogen in children as well as in adults. For children, it is the major cause of otitis media (OM), the most prevalent pediatric bacterial infection. This disease frequently leads to conductive hearing loss and is responsible for nearly 30 million doctor visits each year, resulting in approximately 5 billion dollars for patient care in the United States [2], [3]. In adults, NTHi is also the predominant bacteria linked with the exacerbation of chronic obstructive pulmonary disease (COPD), the fourth leading cause of death in the United States [4], [5]. Antibiotics are the standard method of treating NTHi infections [6]. However, since over 80% of NTHi strains are drug-resistant there is an urgent need for novel therapeutic agents [6], [7].

A classic hallmark of both OM and COPD is the overactive inflammatory response. During an infection, NTHi induces epithelial cells to release numerous proinflammatory chemokines, including IL-8 [8]. IL-8 plays a key role in inflammatory response, primarily by recruiting neutrophils to the site of infection, in order to combat the present pathogen [9]. While IL-8-dependent activity promotes the clearance of the original bacteria, leading to repair and healing, an overactive inflammatory response can result in severe tissue damage to the host, thus causing debilitating diseases such as OM and COPD [10]. Due to the critical role that IL-8 plays in inflammation, it is vital that this chemokine be stringently regulated. We have previously shown that both the MEK/ERK pathway and the NF-κB pathway are necessary for IL-8 expression [11]. However, because NF-κB is ubiquitously and critically involved in all aspects of immune response as well as other biological pathways, manipulation of the system could lead to unwanted side effects. Therefore, we chose to focus our investigation on the regulation of the MEK/ERK pathway. This pathway consists of a series of cell surface receptors, such as epidermal growth factor receptor that relay surface signals on the cell membrane to regulatory components that can direct the cell response, specifically proliferation and regulation of apoptosis [12]. Manipulation of this pathway, by using MEK inhibitors to directly target ERK signaling, has been shown to be both effective and non-toxic, both *in vitro* and *in vivo* [13]–[15].

Recent studies have shown that the deubiquitinase (DUB), cylindromatosis (CYLD), is critical in modulating NTHi-induced inflammation [16], [17]. CYLD is induced by bacterial pathogens, and acts as a negative regulator for various signaling molecules, such as TRAF6 and Akt, by removing lysine 63-linked polyubiquitin chains from targeted proteins [18]–[20]. Recently, we, along with others, have demonstrated that CYLD also inhibits MAPKs including p38 and JNK [21]–[23]. However, it remains unknown if CYLD also suppresses ERK activation induced by bacterial pathogens.

Here, we show that CYLD suppresses NTHi-induced IL-8 expression by specifically targeting the activation of ERK. Interestingly, CYLD suppresses NTHi-induced ERK activation by upregulating another negative regulator, MAP Kinase Phosphatase-1 (MKP-1). Understanding of this mechanism could lead to novel therapies against NTHi infections geared towards inhibiting the detrimental effects of an overactive inflammatory response against NTHi infections.

## Results

### CYLD is a key negative regulator for NTHi-induced IL-8 transcription

**Figure 1:**
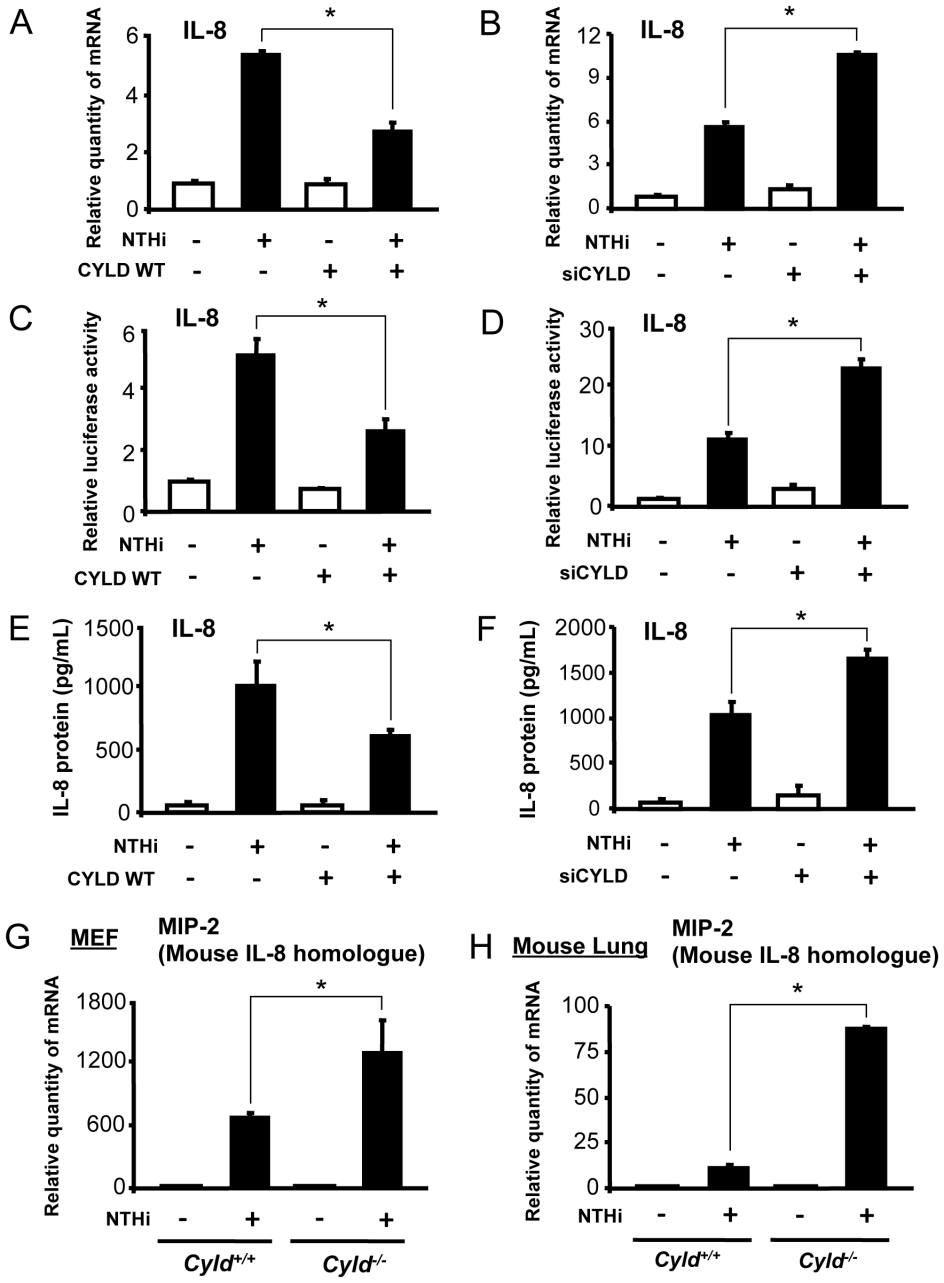
CYLD negatively regulates NTHi-induced IL-8 transcription *in vitro* and *in vivo*. (**A–B**) A549 cells transfected with (**A**) CYLD WT or (**B**) siCYLD were stimulated with NTHi for 5 h, and IL-8 mRNA expression was measured. (**C–D**) A549 cells transfected with IL-8 luciferase reporter gene and (**C**) CYLD WT or (**D**) siCYLD were stimulated with NTHi, and IL-8 transcriptional activity was measured by luciferase assay. (**E–F**) Cells transfected with (**E**) CYLD WT or (**F**) siCYLD were stimulated with NTHi for 12 h, and IL-8 protein in cell culture supernatants was measured by ELISA. (**G**) MIP-2, a mouse homologue of human IL-8, mRNA expression was measured in MEF cells from *Cyld^+/+^* and *Cyld^−/−^* mice stimulated with NTHi for 5 h. (**H**) MIP-2 mRNA expression was measured in lung tissues from *Cyld^+/+^* and *Cyld^−/−^* mice inoculated with NTHi. Data are mean ± SD (*n* = 3). *p<0.05. Statistical analysis was performed using Student's *t*-test. Data are representative of three or more independent experiments.

We first examined the effect of CYLD WT on NTHi-induced IL-8 mRNA expression in human lung epithelial A549 cells *in vitro* by performing Q-PCR analysis. As shown in Fig. 1A, IL-8 induction is markedly inhibited by CYLD WT. Consistent with this result, CYLD knockdown with siRNA-CYLD (siCYLD) significantly increased IL-8 mRNA levels (Fig. 1B). We further confirmed the inhibition of IL-8 transcription by performing a luciferase assay using an IL-8 promoter fused to a luciferase reporter gene. As shown in Fig. 1C and 1D, overexpressing CYLD WT suppressed, whereas siCYLD enhanced IL-8 transcription. Moreover, the effect of CYLD WT and siCYLD on IL-8 protein induction was also confirmed by enzyme-linked immunosorbent assay (ELISA) based on specific anti-IL-8 antibody (Fig. 1E and 1F). Additionally, similar result was also observed in human cervical epithelial HeLa cells (Fig. S1), which further suggests the generalizability of inhibition of IL-8 by CYLD. In addition to human epithelial cells, we also examined the regulation of MIP-2, the mouse homologue of human IL-8, using MEF cells isolated from *Cyld* ^−/−^ mice as well as their littermate control. Interestingly, compared with WT MEF cells, NTHi-induced MIP-2 expression was significantly elevated in *Cyld* ^−/−^ MEF cells (Fig. 1G). We next asked whether this regulatory role of CYLD was also present *in vivo*, in the mouse lung. We measured MIP-2 mRNA levels in the lungs from both WT and *Cyld* ^−/−^ mice 9 h after intratracheal NTHi inoculation. MIP-2 expression was indeed significantly enhanced in *Cyld* ^−/−^ mice compared to WT (Fig. 1H). Taken together, these data demonstrate that CYLD is a critical negative regulator for NTHi-induced IL-8 expression, *in vitro* and *in vivo*.

### CYLD inhibits NTHi-induced activation of ERK

**Figure 2:**
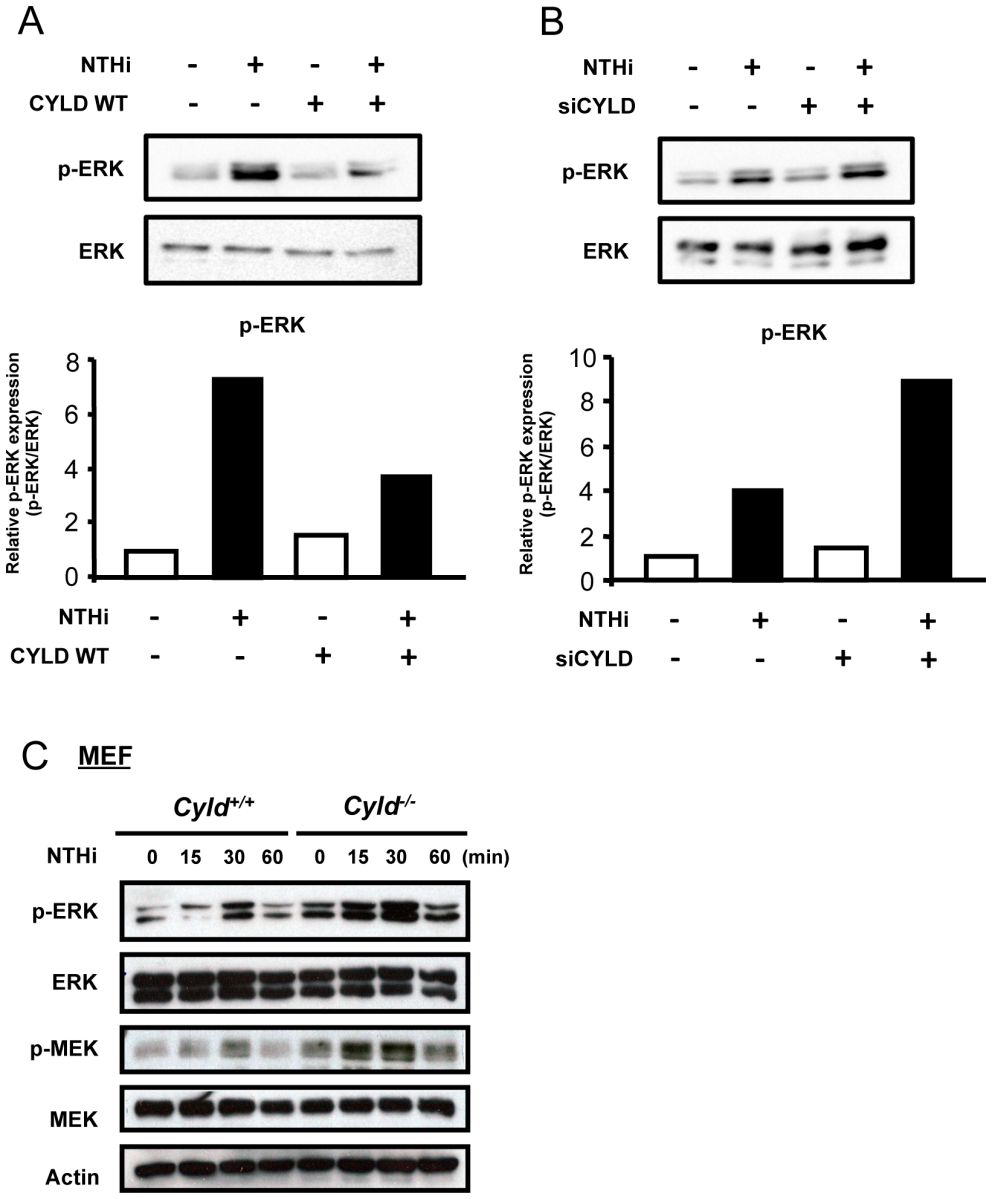
CYLD inhibits NTHi-induced activation of ERK. (**A–B**) Cells transfected with (**A**) CYLD WT or (**B**) siCYLD were stimulated with NTHi for 30 min, and cell lysates were analyzed by immunoblotting with the indicated antibodies (upper panel). Phosphorylated ERK (pERK) protein expression was quantified (lower panel). (**B**) Cells transfected with siCON or siCYLD were stimulated with NTHi for 30 min, and cell lysates were analyzed by immunoblotting with the indicated antibodies. (**C**) MEF cells from *Cyld^+/+^* and *Cyld^−/−^* mice stimulated with NTHi for various times as indicated in the figure, and cell lysates were analyzed by immunoblotting with the indicated antibodies. Data are representative of three or more independent experiments.

Having demonstrated that CYLD suppresses NTHi-induced IL-8 expression, we next aimed to investigate the mechanism through which this regulation occurs. The ERK signaling pathways have been shown to play an important role in controlling inflammatory responses [29]. We previously showed that ERK pathways are critical for the upregulation of IL-8 [11]. We thus asked whether CYLD inhibited ERK activation. As shown in Fig. 2A, CYLD WT inhibited ERK phosphorylation, while CYLD knockdown, via siCYLD, markedly enhanced ERK activation by NTHi (Fig. 2B). Moreover, phosphorylation of ERK and MEK, a key upstream activator for ERK, is notably higher in *Cyld* ^−/−^ MEF cells compared with their WT controls (Fig. 2C). Collectively, it is evident that CYLD specifically inhibits the activation of ERK induced by bacterial pathogen in both human and mouse cells.

### CYLD negatively regulates NTHi-induced IL-8 expression via inhibition of the ERK pathway

**Figure 3:**
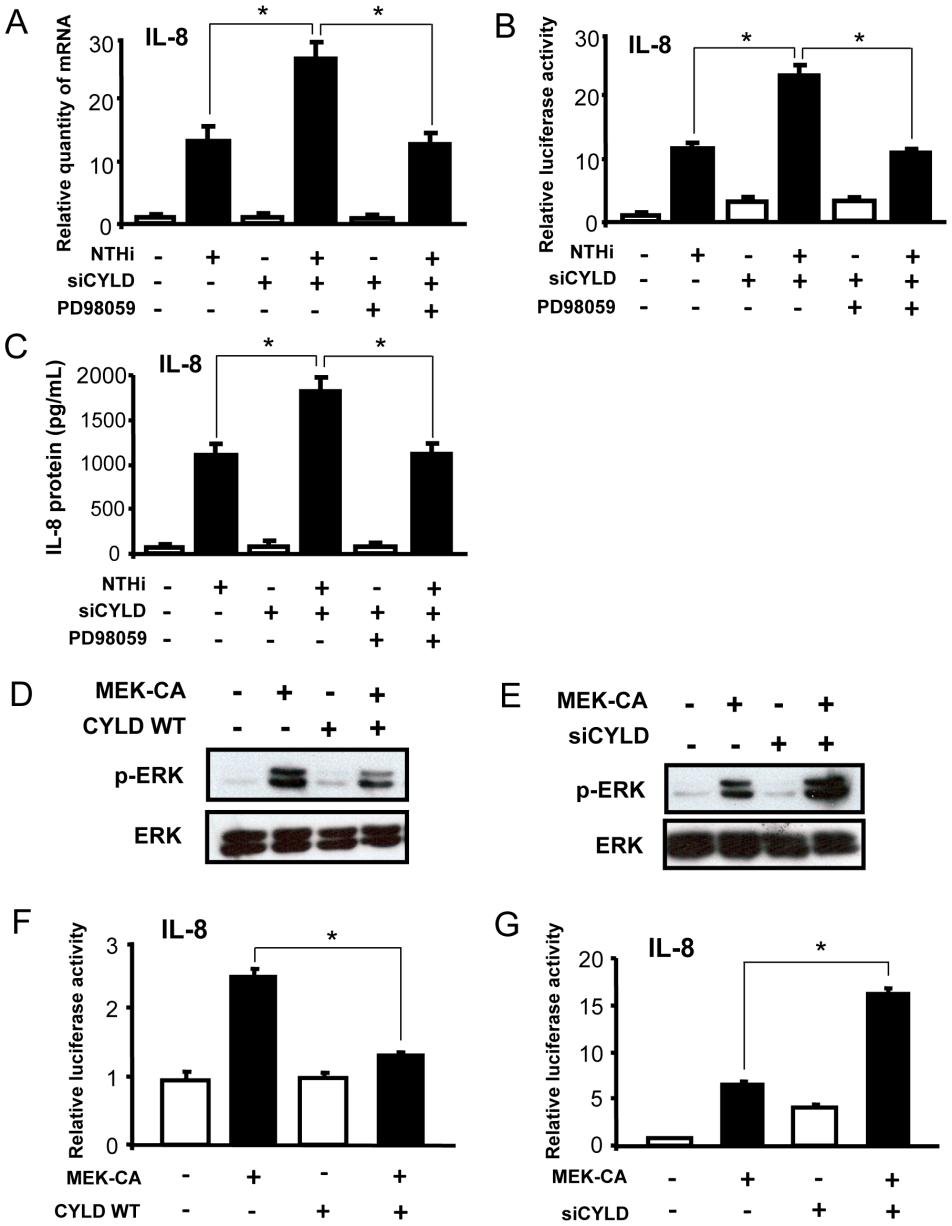
CYLD negatively regulates NTHi-induced IL-8 expression via inhibition of ERK pathway. (**A**) A549 cells transfected with siCYLD were pretreated with PD98059 (20 µM) for 1 h, and IL-8 mRNA expression was measured after NTHi-stimulation. (**B**) Cells transfected with IL-8 luciferase reporter gene and siCYLD were pre-treated with PD98059 (20 µM) for 1 h, followed by NTHi-stimulation, and IL-8 transcriptional activity was measured by luciferase assay. (**C**) Cells transfected with siCYLD were pretreated with PD98059 (20 µM) for 1 h, followed by NTHi-stimulation, and IL-8 protein in the supernatants of cells was measured by ELISA. (**D–E**) Cells were transfected with MEK-CA and (**D**) CYLD WT or (**E**) siCYLD, and cell lysates were analyzed by immunoblotting with the indicated antibodies. (**F–G**) Cells were transfected with IL-8 luciferase reporter gene, MEK-CA and (**F**) CYLD WT or (**G**) siCYLD, and IL-8 transcriptional activity was measured. Data in **A–C, F** and **G** are mean ± SD (*n* = 3). *p<0.05. Statistical analysis was performed using Student's *t*-test. MEK-CA, constitutive active form of MEK. Data are representative of three or more independent experiments.

To further elucidate the role of CYLD in IL-8 levels, we used the specific MEK inhibitor, PD98059, to examine the involvement of the ERK pathway in CYLD-mediated suppression of IL-8. As shown in Fig. 3A, 3B, and 3C, PD98059 reversed the enhancement of NTHi-induced IL-8 expression at the transcription, mRNA and protein levels by siCYLD. We next sought to determine whether CYLD inhibits upregulation of IL-8 expression induced by direct activation of ERK using a constitutively active form of MEK (MEK-CA). As shown in Fig. 3D, 3E and Fig. S2A, CYLD WT reduced, whereas siCYLD enhanced, activation of ERK induced by overexpression of MEK-CA. Moreover, overexpression of CYLD WT suppressed, whereas CYLD knockdown by siCYLD enhanced, MEK-CA-induced IL-8 expression (Fig. 3F, 3G and Fig. S2B). Together, these data suggest that CYLD negatively regulates IL-8 expression by specifically targeting the ERK signaling pathway.

### CYLD is a positive regulator of NTHi-induced upregulation of MKP-1 expression

**Figure 4:**
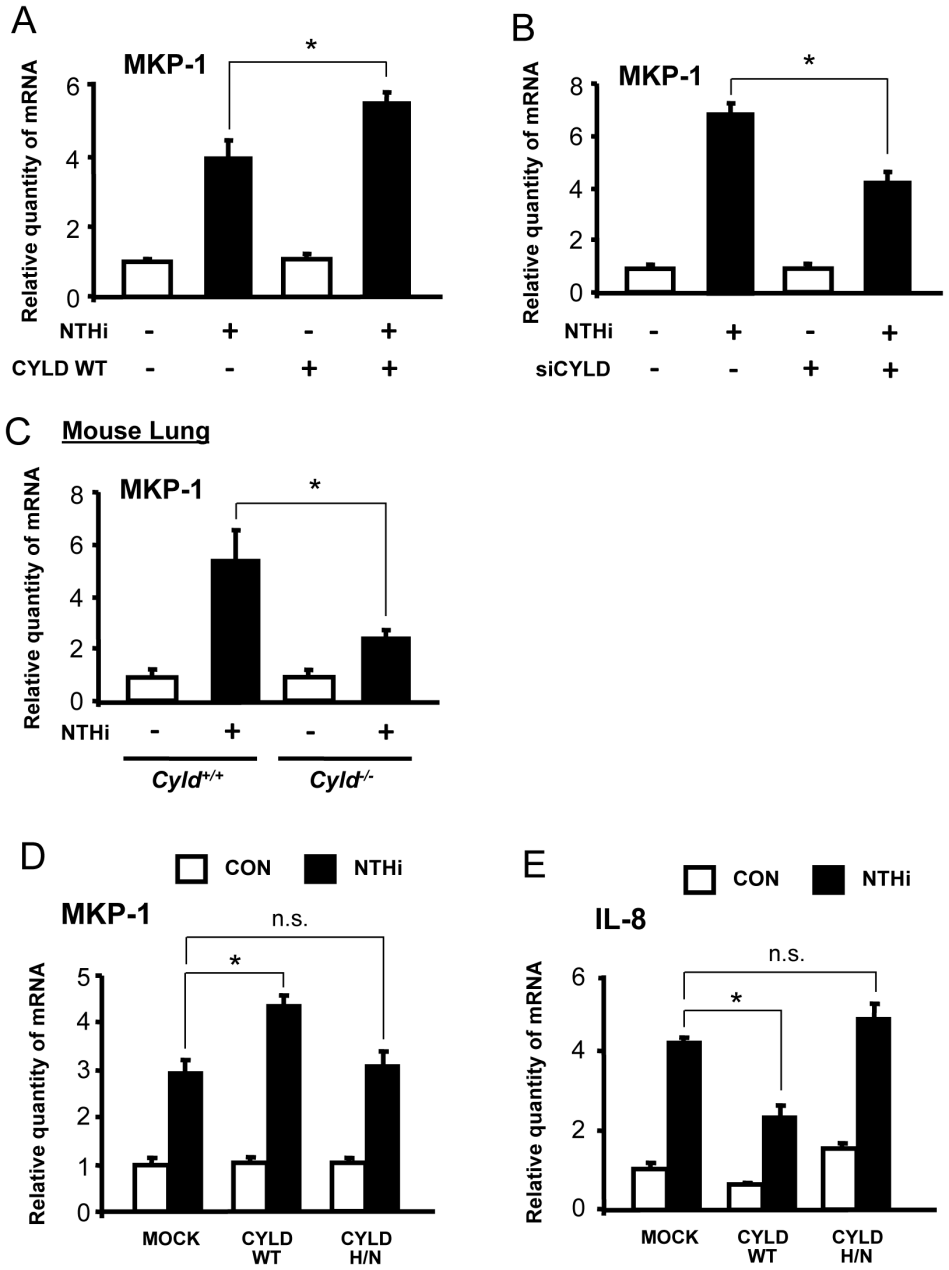
CYLD is a positive regulator of NTHi-induced upregulation of MKP-1 expression. (**A–B**) Cells transfected with (**A**) CYLD WT or (**B**) siCYLD were stimulated with NTHi for 1 h, and MKP-1 mRNA expression was measured. (**C**) MKP-1 mRNA expression was measured in lung tissues from *Cyld^+/+^* and *Cyld^−/−^* mice inoculated with NTHi. (**D**) Cells transfected with CYLD WT or DUB activity-deficient CYLD mutant (CYLD H/N) were stimulated with NTHi for 1 h, and MKP-1 mRNA expression was measured. (**E**) Cells transfected with CYLD WT or CYLD H/N were stimulated with NTHi for 5 h, and IL-8 mRNA expression was measured. Data are mean ± SD (*n* = 3). *p<0.05. Statistical analysis was performed using Student's *t*-test. n.s., nonsignificant. Data are representative of three or more independent experiments.

We next sought to determine how CYLD negatively regulates the ERK signaling pathway in order to mediate NTHi-induced IL-8 expression. MAP Kinase Phophatase-1 (MKP-1) has been shown to be a key inactivator of ERK via dephosphorylation [30]. Thus, we investigated whether CYLD positively regulated NTHi-induced MKP-1 expression. Interestingly, the overexpression of CYLD WT enhanced (Fig. 4A), whereas siCYLD suppressed, MKP-1 mRNA levels (Fig. 4B). In addition to human epithelial cells, we also measured MKP-1 mRNA levels in the lungs from both WT and *Cyld* ^−/−^ mice 4 h after intratracheal NTHi inoculation. MKP-1 expression was indeed significantly reduced in *Cyld* ^−/−^ mice compared to WT (Fig. 4C). Taken together, our data suggest that CYLD mediates NTHi-induced upregulation of MKP-1.

We next sought to determine how CYLD positively regulates MKP-1 expression. Because CYLD has been identified as a DUB, we investigated whether DUB activity is required for the CYLD-mediated upregulation of MKP-1 induced by NTHi. As shown in Fig. 4D and 4E, DUB activity-deficient CYLD mutant (CYLD H/N) had no effect on NTHi-induced MKP-1 and IL-8 expression, whereas overexpression of CYLD WT enhanced MKP-1 induction and suppressed IL-8 expression. Thus, our data demonstrate that CYLD upregulates MKP-1 expression in a DUB activity-dependent manner.

### CYLD negatively regulates NTHi-induced IL-8 expression via upregulating MKP-1

**Figure 5:**
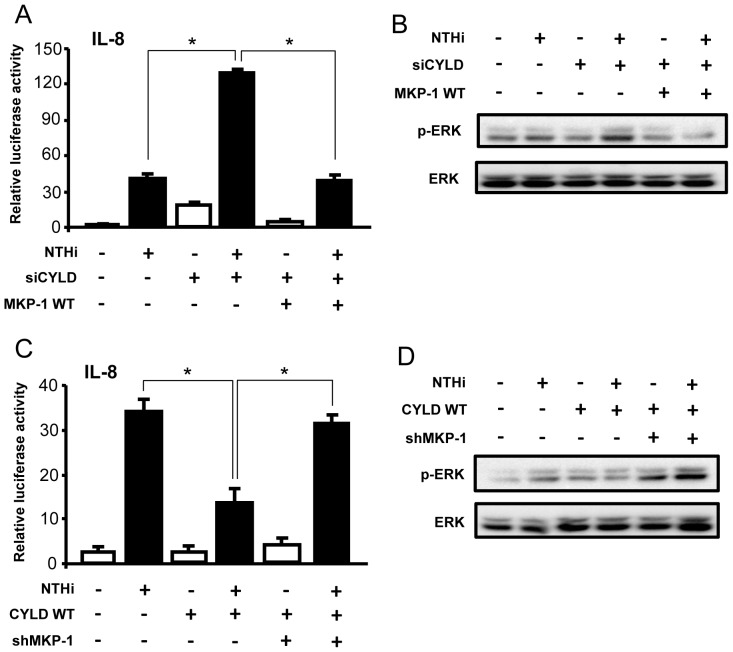
CYLD negatively regulates NTHi-induced IL-8 expression via upregulating MKP-1. (**A**) Cells transfected with IL-8 luciferase reporter gene, siCYLD and MKP-1 WT were stimulated with NTHi for 5 h, and IL-8 transcriptional activity was measured. (**B**) Cells transfected with siCYLD and MKP-1 WT were stimulated with NTHi for 30 min, and cell lysates were analyzed by immunoblotting with the indicated antibodies. (**C**) Cells transfected with IL-8 luciferase reporter gene, CYLD WT and shMKP-1 were stimulated with NTHi for 5 h, and IL-8 transcriptional activity was measured. (**D**) Cells transfected with CYLD WT and shMKP-1 were stimulated with NTHi for 30 min, and cell lysates were analyzed by immunoblotting with the indicated antibodies. Data in **A** and **C** are mean ± SD (*n* = 3). *p<0.05. Statistical analysis was performed using Student's *t*-test. Data are representative of three or more independent experiments.

**Figure 6:**
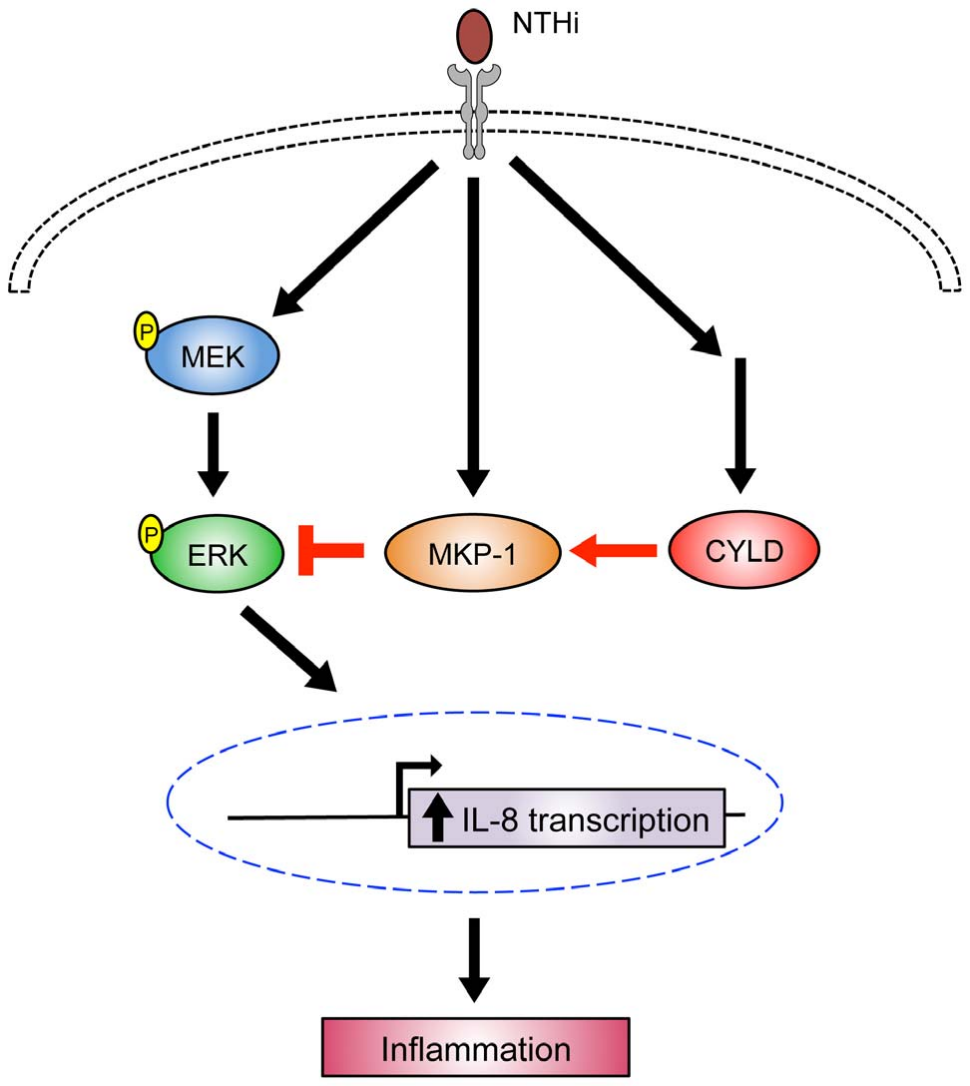
Schematic model illustrating that CYLD negatively regulates NTHi-induced IL-8 expression via MKP-1-dependent inhibition of ERK. As indicated, CYLD negatively regulates NTHi-induced IL-8 expression through MEK/ERK signaling pathway by upregulating phosphatase MKP-1.

Having shown that CYLD negatively regulates NTHi-induced IL-8 expression via the inhibition of ERK and positively regulates MKP-1, we next investigated whether CYLD acts as a negative regulator for ERK-dependent IL-8 induction via MKP-1. Indeed, we found that overexpression of MKP-1 WT reversed the enhancement of IL-8 transcription and ERK phosphorylation by siCYLD (Fig. 5A and 5B). Furthermore, MKP-1 knockdown by MKP-1 short hairpin RNA (shMKP-1) counteracted the inhibitory effect of CYLD WT on IL-8 transcription and ERK phosphorylation (Fig. 5C and 5D). Collectively, our data suggest that CYLD negatively regulates NTHi-induced IL-8 expression via a MKP-1-dependent inhibition of ERK pathway (Fig. 6).

## Discussion

OM and COPD are both debilitating diseases caused by NTHi, in children and adults, respectively. A classic hallmark of both is the overactive host inflammatory response. In this study, we demonstrated that CYLD strongly suppressed NTHi-induced IL-8 expression in human lung epithelial cells. Concurrent with this data, we also showed that the mouse homologue of IL-8, MIP-2, was also significantly upregulated in *Cyld* ^−/−^ mouse lungs infected with live NTHi. Collectively, these data suggest that CYLD is a critical suppressor of IL-8 expression. We further demonstrated that CYLD decreased IL-8 levels via inactivation of the ERK signaling pathway. Importantly, we determined that CYLD negatively regulates ERK-dependent IL-8 expression specifically by inducing MKP-1 expression. Our studies thus provide a novel insight into the tight regulation of bacteria-induced inflammation by CYLD and may lead to the development of new therapeutic strategy.

The finding that CYLD may target the ERK pathway in negatively controlling IL-8 expression is particularly significant. In recent years, the role of CYLD as a negative regulator of inflammation has been actively explored. ERK is widely involved in cellular activities, including survival, proliferation, differentiation, and death [33]. Recently, it was shown as an important regulator in the host inflammatory response [29], [34]. Known targets of CYLD include TRAF2, TRAF6, NEMO, Akt, JNK, Smad7, TAK1, Bcl3 and RIP1 [19], [20], [31], [32]. However, it remained largely unknown whether or how CYLD modulated ERK signaling, when induced by bacterial pathogens. Our study revealed a previously unexplored role of CYLD in regulating an ERK-dependent inflammatory response. Here we provide clear evidence that CYLD inhibits NTHi-induced activation of ERK (Fig. 2), and we also demonstrate that CYLD controls IL-8 expression via specifically targeting ERK phosphorylation by activating the ERK pathway with a constitutively active form of MEK (Fig. 3). Recent studies have shown that the DUB activity in CYLD is required for inactivating target proteins [20], [35]–[38]. Future studies will focus on using DUB activity-deficient CYLD mutant constructs to determine whether DUB activity is essential for the CYLD-mediated suppression of ERK signaling pathway induced by bacterial pathogens.

In addition, we further investigated the detailed mechanism by which CYLD negatively regulates the ERK signaling pathway in a DUB activity-dependent or independent manner. In this study, we focused on the critical negative regulator of ERK, MAP Kinase Phosphatase-1 (MKP-1). MKP-1 is a well-defined inactivator of ERK that acts through dephosphorylation [30], [39]. While the regulation of MKP-1 has been extensively studied [39], [40], it is still relatively unknown if CYLD regulates MKP-1 expression. For the first time, we provided direct evidence that CYLD positively regulates NTHi-induced MKP-1 expression in a DUB activity-dependent manner (Fig. 4). This upregulation of MKP-1 further suppressed NTHi-induced ERK activation (Fig. 5). This finding is of particular translational interest and significance, as the induction of negative regulators is potentially an attractive therapeutic strategy for effectively controlling overactive inflammation without causing adverse side effects [41], [42]. Therefore, investigating the molecular mechanisms of how CYLD upregulates MKP-1 may bring new insights into the tight regulation of inflammatory responses. Our future investigation will focus on elucidating which signaling molecules are involved in CYLD-mediated upregulation of MKP-1.

In conclusion, our study unveiled a novel mechanism underlying the negative regulation of ERK-dependent expression of pro-inflammatory mediators by CYLD. Future studies may provide a unique opportunity to design new therapies to stringently regulate inflammation by focusing on the key negative regulators of inflammation. As this research proceeds, it may have a broader impact by applying these insights to treat other bacterial infections, various forms of cancer, and neurodegenerative disorders controlled by the ERK pathway such as Huntington's, Alzheimer's and Parkinson's diseases [33], [43], [44].

## Materials and Methods

### Reagents and antibodies

PD98059 was purchased from Enzo Life Sciences. Antibodies against phospho-ERK1/2 (Thr-202/Tyr-204), total ERK1/2, phospho-MEK1/2 (Ser-217/Ser-221) and total MEK1/2 were purchased from Cell Signaling Technology. Antibody against β-actin was purchased from Santa Cruz Biotechnology.

### Cell culture

A549 and HeLa cells were maintained in F-12K medium (Gibco) and DMEM (Cellgro), and supplemented with 10% FBS (Sigma-Aldrich) and 100 U/mL penicillin and 100 µg/mL streptomycin (Gibco). Wild-type (WT) and *Cyld^−/−^* MEF cells were maintained in DMEM and obtained as previously described [20], [21]. All cells were cultured at 37°C in a humidified atmosphere of 5% CO_2_.

### Bacterial strains and culture condition

NTHi strain 12, a clinical isolate, was grown overnight on chocolate agar plate in an atmosphere of 5% CO_2_ at 37°C. The bacteria were then inoculated in brain heart infusion (BHI) broth enriched with 3.5 µg/ml NAD and hemin. Following overnight incubation, bacteria were subcultured into 5 ml of fresh BHI broth and grown to log phase, as determined by measuring optical density value. NTHi were then washed and suspended in phosphate buffered saline (PBS) for *in vitro* cell experiments and in isotonic saline for *in vivo* animal experiments.

### Real-time quantitative RT-PCR analysis

Total RNA was isolated using TRIzol reagent (Invitrogen), as per the manufacturer's instructions. TaqMan reverse transcription reagents (Applied Biosystems) were used for the synthesis of complementary DNA from total RNA, as described [22], [24], [25]. Real-time quantitative PCR was performed using SYBR Green Universal Master Mix (Applied Biosystems) and an ABI 7500 sequence detector, along with the manufacturer's software (7000v1.3.1; Applied Biosystems), as previously described [24]. Relative quantities of mRNAs were calculated using the comparative threshold cycle method and normalized using human cyclophilin and mouse glyceraldehydes-3-phosphate dehydrogenase (GAPDH) as an endogenous control. The primers for human IL-8 and cyclophilin, as well as, mouse MIP-2 and GAPDH were previously described [16], [24]. The primer sequences for human and mouse MKP-1 are as follows: human MKP-1: 5′-GCTGTGCAGCAAACAGTCGA-3′ and 5′-GCCACCCTGATCGTAGAGTG-3′; mouse MKP-1: 5′-GCTGTGCAGCAAACAGTCGA-3′ and 5′-CGATTAGTCCTCATAAGGTA-3′

### Plasmids, transfections, and luciferase assay

The expression plasmids Flag-WT-CYLD, Flag-H/N-CYLD, WT-MKP1 and luciferase reporter construct of IL-8 were previously described [11], [20], [26]. The constitutively active form of MEK (MEK-CA) was kindly provided by Dr. Saltiel (Life Sciences Institute, University of Michigan, Ann Arbor, MI). In all experiments, an empty vector was used as a control. All transient transfections were performed using TransIT-LT1 reagent (Mirus) according to manufacturer's instructions. For experiments using inhibitors, the transfected cells were pretreated with or without chemical inhibitors for 1 h followed by 5 h incubation with NTHi. Transcriptional activity of reporter gene was measured by luciferase assay and normalized with respect to β-galactosidase activity as described previously [16], [27].

### RNA-mediated interference

CYLD expression was mediated via RNA-mediated interference using pSUPER-CYLD, as previously described [24]. The sequence for siCYLD is 5′-GATCCCCGAGCTACTGAGGACAGAAATTCAAGAGATTTCTGTCCTCAGTAGCTCTTTTTGGAAA-3′. To generate MKP-1 knockdown construct (shMKP-1), oligonucleotide encoding short hairpin transcript corresponding to MKP-1 was cloned into pSUPER. Knockdown of MKP-1 using shMKP-1 was performed using TransIT-LT1 reagent (Mirus) following the manufacturer’s instruction. The target sequence for cloning shMKP-1 is 5′-CTGCCTTGATCAACGTCTC-3′.

### Western blot analysis

Western blot analyses were performed as previously described [28]. Cells were treated with NTHi for 30 min. Whole-cell lysate was separated in a 10% SDS-PAGE gel, transferred to polyvinylidene fluoride membrane, then incubated with antibodies against phospho-ERK1/2, total ERK1/2, phospho-MEK1/2, total MEK1/2, or β-actin. Respective proteins were visualized by using secondary HRP-conjugated rabbit or mouse IgG antibody (Santa Cruz Biotechnology) and the ECL detection system (Amersham ECL Prime Western Blotting Detection Regent, GE Healthcare).

### Enzyme-linked immunosorbent assay (ELISA)

Human IL-8 protein in the supernatants of cells was measured with LEGEND MAX Human IL-8 ELISA kit (BioLegend, Inc.) according to the manufacturer's instructions. Cells were treated with NTHi when they reached 80% confluence. Cell culture supernatants were collected 12 h after NTHi treatment, and centrifuged to remove debris prior to the assay.

### Mice and animal experiments

*Cyld^−/−^* mice, in a C57BL/6J background, were previously described [24]. Age-matched WT mice were used as controls. To determine NTHi-induced lung inflammation, mice were intratracheally inoculated at a concentration of 5×10^7^ CFU per mouse, while saline was utilized as a control. The mice were sacrificed 9 h (for MIP-2) or 4 h (for MKP-1) after bacterial inoculation and the lungs were harvested for total RNA extraction, as previously described [24]. All animal experiments were approved by the Institutional Animal Care and Use Committee at Georgia State University.

### Statistical analysis

All experiments were repeated in at least three independent experiments. Data are shown as mean ± SD of *n* determinations. Statistical analysis was assessed by two-tailed unpaired student's *t*-test. *p*<0.05 was considered statistically significant.

## Supporting Information

Figure S1 **The effects of CYLD WT and siCYLD on NTHi-induced IL-8 expression in HeLa.** (**A–B**) HeLa cells transfected with (**A**) CYLD WT or (**B**) siCYLD were stimulated with NTHi for 5 h, and IL-8 mRNA expression was measured. (**C–D**) HeLa cells transfected with IL-8 luciferase reporter gene and (**C**) CYLD WT or (**D**) siCYLD were stimulated with NTHi, and IL-8 transcriptional activity was measured by luciferase assay. Data are mean ± SD (*n* = 3). *p<0.05. Statistical analysis was performed using Student's *t*-test. Data are representative of three or more independent experiments.
(TIF)

Figure S2 **The effects of constitutively active form of MEK (MEK-CA).** (**A**) Cells were transfected with various amount of MEK-CA, and cell lysates were analyzed by immunoblotting with the indicated antibodies. (**B**) Cells were transfected with IL-8 luciferase reporter gene and various amount of MEK-CA, and IL-8 transcriptional activity was measured by luciferase assay. Data in **B** are mean ± SD (*n* = 3). *p<0.05. Statistical analysis was performed using Student's *t*-test. Data are representative of three or more independent experiments.
(TIF)
