# Supplementary figures and images for: CYLD Negatively Regulates Nontypeable Haemophilus influenzae-Induced IL-8 Expression via Phosphatase MKP-1-Dependent Inhibition of ERK
Source: PLoS One. 2014 Nov 12;9(11):e112516. doi: 10.1371/journal.pone.0112516 (PMC4229244; doi:10.1371/journal.pone.0112516)

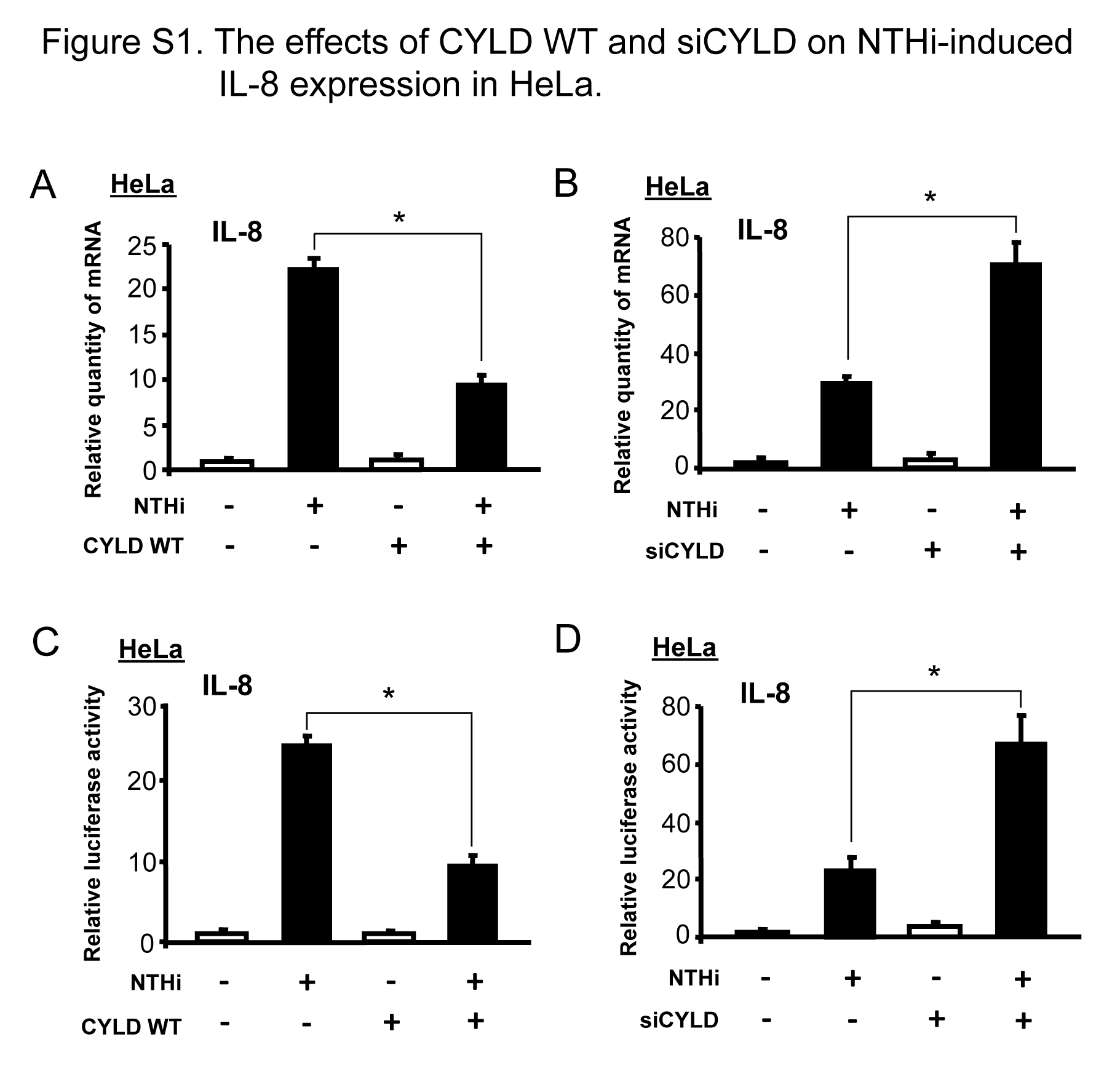

Supplement: Figure S1 — The effects of CYLD WT and siCYLD on NTHi-induced IL-8 expression in HeLa. (A–B) HeLa cells transfected with (A) CYLD WT or (B) siCYLD were stimulated with NTHi for 5 h, and IL-8 mRNA expression was measured. (C–D) HeLa cells transfected with IL-8 luciferase reporter gene and (C) CYLD WT or (D) siCYLD were stimulated with NTHi, and IL-8 transcriptional activity was measured by luciferase assay. Data are mean ± SD (n = 3). *p<0.05. Statistical analysis was performed using Student's t-test. Data are representative of three or more independent experiments. (TIF) [file pone.0112516.s001.tif]

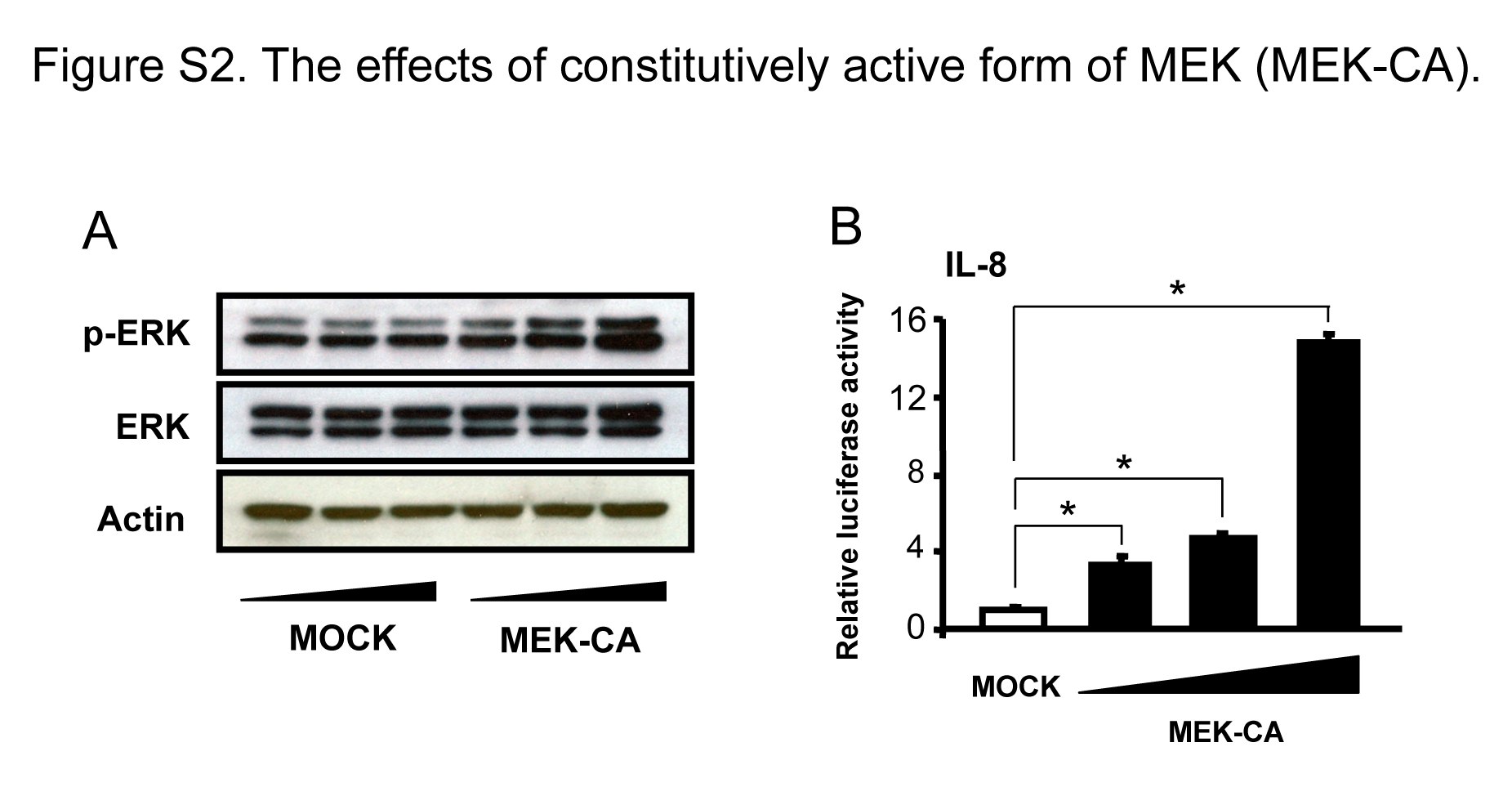

Supplement: Figure S2 — The effects of constitutively active form of MEK (MEK-CA). (A) Cells were transfected with various amount of MEK-CA, and cell lysates were analyzed by immunoblotting with the indicated antibodies. (B) Cells were transfected with IL-8 luciferase reporter gene and various amount of MEK-CA, and IL-8 transcriptional activity was measured by luciferase assay. Data in B are mean ± SD (n = 3). *p<0.05. Statistical analysis was performed using Student's t-test. Data are representative of three or more independent experiments. (TIF) [file pone.0112516.s002.tif]
